# Neuroprotective Properties of Kempferol Derivatives from *Maesa membranacea* against Oxidative Stress-Induced Cell Damage: An Association with Cathepsin D Inhibition and PI3K/Akt Activation

**DOI:** 10.3390/ijms221910363

**Published:** 2021-09-26

**Authors:** Danuta Jantas, Janusz Malarz, Thanh Nguyen Le, Anna Stojakowska

**Affiliations:** 1 Maj Institute of Pharmacology, Polish Academy of Sciences, Smętna Street 12, 31-343 Kraków, Poland; jantas@if-pan.krakow.pl (D.J.); malarzj@if-pan.krakow.pl (J.M.); 2 Institute of Marine Biochemistry, Graduate University of Science and Technology, Vietnam Academy of Science and Technology, 18 Hoang Quoc Viet, Caugiay, Hanoi 1000000, Vietnam; lethanh@imbc.vast.vn

**Keywords:** flavonols, hydrogen peroxide, isoquercitrin, kaempferitrin, *Maesa*, 6-OHDA, Primulaceae, polyphenols, α-rhamnoisorobin, SH-SY5Y cells

## Abstract

As components of the human diet with potential health benefits, flavonols are the subject of numerous studies, confirming their antioxidant, free radical scavenging and anti-inflammatory activity. Taking into consideration the postulated pathogenesis of certain CNS dysfunctions characterized by neuronal degradation, flavonols may prevent the decay of neurons in multiple pathways. Leaves of *Maesa membranacea* yielded several flavonol glycosides including α-rhamnoisorobin (kaempferol 7-*O-*α-rhamnoside) and kaempferitrin (kaempferol 3,7-di-*O*-α-rhamnoside). The latter compound was a major constituent of the investigated plant material. Neuroprotective effects of kaempferitrin and α-rhamnoisorobin were tested in vitro using H_2_O_2_-, 6-OHDA- and doxorubicin-induced models of SH-SY5Y cell damage. Both undifferentiated and differentiated neuroblastoma cells were used in the experiments. α-Rhamnoisorobin at a concentration range of 1–10 µM demonstrated cytoprotective effects against H_2_O_2_-induced cell damage. The compound (at 1–10 µM) was also effective in attenuating 6-OHDA-induced neurotoxicity. In both H_2_O_2_- and 6-OHDA-induced cell damage, kaempferitrin, similar to isoquercitrin, demonstrated neuroprotective activity at the highest of the tested concentrations (50 µM). The tested flavonols were not effective in counteracting doxorubicin-induced cytotoxicity. Their caspase-3- and cathepsin D-inhibitory activities appeared to be structure dependent. Inhibition of the PI3-K/Akt pathway abolished the neuroprotective effect of the investigated flavonols.

## 1. Introduction

Flavonols, derivatives of 3-hydroxy-2-phenyl-4H-chromen-4-one, form a subclass of flavonoids—plant polyphenols of common occurrence. The group of plant-specialized metabolites demonstrates a wide array of biological activities, including anti-inflammatory, antioxidative and free radical-scavenging and comprises compounds of different substitution patterns, often in their glycosidic forms. The consequence of this structural diversity is the difference in activity profile, bioavailability and cytotoxicity of individual compounds. As ubiquitous constituents of plants, including plant foods, flavonols have been extensively studied in respect to their potential benefits or risks to human health. The most commonly found plant metabolites of this type are quercetin, kaempferol and their corresponding 3-*O*-glucosides-isoquercitrin and astragalin. Pharmacological activities of the compounds and their role as the components of human diet are the subject of numerous studies [1,2,3,4,5]. Recently, two review papers summarizing the research on neuroprotective action of kaempferol, quercetin and glycosidic derivatives of the compounds were published [6,7]. Kaempferol 7-*O*-rhamnosides, however, remain a poorly investigated group of kaempferol derivatives in regard of their neuroprotective potential.

Numerous intracellular signaling pathways may be involved in the neuroprotection exerted by flavonols [7]. The relationships between the structure of the flavonol molecule and its specificity in targeting particular signaling pathways are far from being completely explored.

The genus *Maesa* comprises over 30 species from tropical areas of the Old World [8]. Plants of the genus have been used in traditional medicine systems of both Africa and Southeast Asia [9,10,11,12,13], mainly as anthelminthic and antiviral remedies. Preparations from roots of *M. lanceolata* Forsk. were taken by Masai as nerve -stimulants [11]. Tea prepared from the leaves of the plant was used in Madagascar as a memory restorer [10]. *M. membranacea* A. DC. (synonym: *M. subrotunda* C.Y. Wu & C. Chen; Primulaceae, Myrsinoideae), a 2–5 m tall shrub growing wild in Cambodia, China, and Vietnam [14,15], is mentioned by the recent ethnobotanical study [16] as one of the 16 plant species with the highest cultural importance for local communities in northern and central Vietnam. Data on polyphenols from *Maesa* spp. are sparse. Manguro et al. [17] described isolation of quercetin, myricetin, and their mono-, di- and triglycosides from a methanolic extract of *M. lanceolata* leaves. Quercetin, rutin (quercetin 3-*O*-rutinoside) and catechin were detected in fruits of *M. indica* (Roxb.) A. DC. [18]. Only two flavonoids, (-)-epicatechin and kaempferol, have been previously isolated from the stems of *M. membranacea* [19]. A preliminary chromatographic analysis of a hydroalcoholic extract from leaves of *M. membranacea* revealed the presence of flavonol glycosides as its major constituents. Further phytochemical work led to the isolation of several phenolic compounds including kaempferol glycosides.

The present study was aimed to investigate kaempferol glycosides isolated from the leaves of *M. membranacea* with particular interest in their neuroprotective activity and its molecular mechanism of action. Isoquercitrin (IQ), frequently studied quercetin glucoside with known neuroprotective properties, was used as a reference compound.

## 2. Results

### 2.1. Phenolic Compounds from the Leaves of M. membranacea

**Figure 1:**
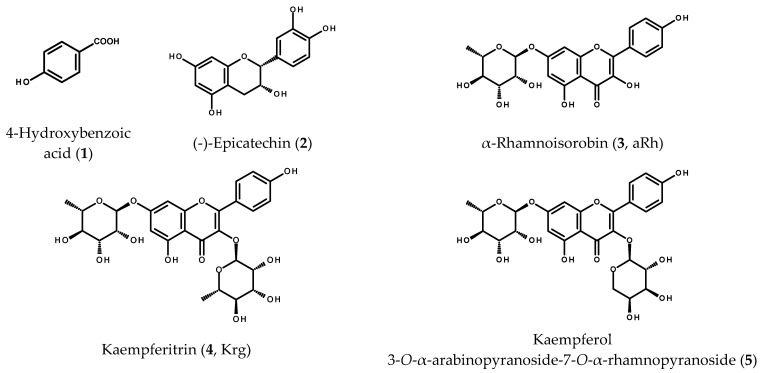
Chemical structures of phenolics **1**–**5** from the leaves of *Maesa membranacea*.

Chromatographic separation of the ethyl acetate subfraction from the hydroalcoholic extract of *M. membranacea* leaves gave fractions rich in phenolic compounds. The fractions that contained the individual compounds or simple mixtures (as shown by HPLC/DAD) were subjected to ^1^H-NMR spectroscopic analysis. On the basis of the obtained spectra and their comparison with the data available from the literature, the purified compounds were identified as known plant constituents (see Figure 1): 4-hydroxybenzoic acid (**1**) [20], (-)-epicatechin (**2**) [21], kaempferol 7-*O*-α-rhamnopyranoside (**3**, α-rhamnoisorobin, aRh), kaempferol 3,7-di-*O*- α-rhamnopyranoside (**4**, kaempferitrin, Krg) and a mixture of kaempferitrin with kaempferol 3-*O*-α-arabinopyranoside-7-*O*-α-rhamnopyranoside (**5**) [22,23]. The mixture of kaempferol diglycosides was not further separated as the proton signals could be easily assigned to the respective known compounds.

Kaempferitrin—the major constituent of the analyzed plant material and kaempferol 3-O-α-arabinopyranoside-7-*O*-α-rhamnopyranoside were quantified in the 70% methanolic extract and in the infusion prepared from leaves of *M. membranacea.* The quantification was performed using the HPLC/DAD method. The contents of kaempferol diglycosides were calculated as percentages of the dry weight of leaves. The Krg content measured in the hydroalcoholic extract and in the infusion was 0.89 ± 0.08% and 0.7 ± 0.02%, respectively. The content of kaempferol 3-*O*-α-arabinopyranoside-7-*O*-α-rhamnopyranoside reached 0.42 ± 0.01% in the 70% methanol extract and 0.31 ± 0.01% in the infusion prepared from the dry plant material.

### 2.2. The Impact of the Tested Flavonols on Cell Proliferation and Biosafety Issues

**Figure 2:**
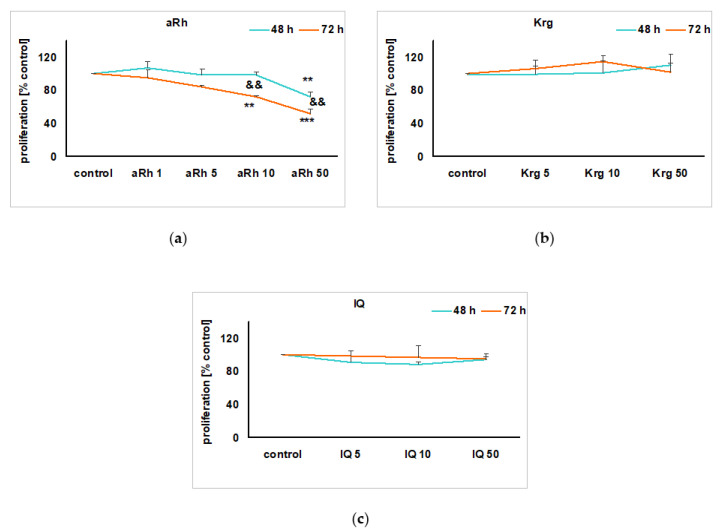
The impact of the tested flavonols on SH-SY5Y cells proliferation: (**a**) α-rhamnoisorobin (aRh); (**b**) kaempferitrin (Krg); (**c**) isoquercitrin (IQ). The cells were seeded at density of 1 × 10^4^ cells/well, in 96-well plates in DMEM containing 10% FBS, and after 24 h were treated either with vehicles or with aRh (1–50 µM), Krg (5–50 µM) and IQ (5–50 µM) for the next 48 and 72 h. Data from MTT reduction assay were normalized to the vehicle-treated cells for the particular time point and are presented as the mean ± SEM from two independent experiments with three repetitions. Two-way ANOVA were used for statistical analysis, ** *p* < 0.01 and *** *p* < 0.001 vs. vehicle treated cells, ^&&^ *p* < 0.01 72 h vs. 48 h.

**Figure 3:**
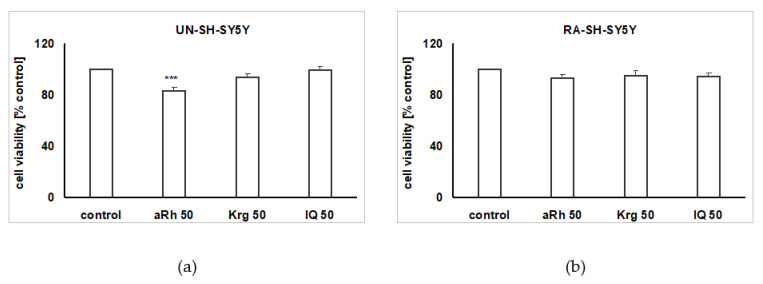
Biosafety assessment of α-rhamnoisorobin (aRh), kaempferitrin (Krg), and isoquercitrin (IQ) in: (**a**) undifferentiated (UN-) and (**b**) retinoic acid-differentiated (RA-) SH-SY5Y cells. The cells were treated either with vehicles or with aRh (50 µM), Krg (50 µM) and IQ (50 µM) for 24 h. Statistical analysis was done with one-way ANOVA. Cell viability was measured with WST-1 assay and the data were normalized to vehicle-treated cells and presented as the mean ± SEM from 4–5 independent experiments with three repetitions. *** *p* < 0.001 vs. vehicle-treated cells.

Under the cell proliferation favoring conditions (culture medium with 10% FBS), we did not notice any impact of Krg or isoquercitrin (IQ) given in wide range of concentrations (5–50 µM), for 48 and 72 h, on the undifferentiated UN-SH-SY5Y cell proliferation rate (Figure 2b,c). However, aRh at concentrations of 10 and 50 µM in a time- and concentration-dependent manner reduced cell proliferation in UN-SH-SY5Y cells by about 28% for 50 µM of aRh after 48 h, and by 30% and 50% after 72 h for 10 and 50 µM aRh, respectively (Figure 2a). After 24 h treatment of UN-SH-SY5Y or retinoic acid-differentiated RA-SH-SY5Y cells with the highest tested concentration of Krg or aRh (50 µM each), we did not find any significant changes in the cell viability when compared to the vehicle-treated cells (Figure 3a,b). aRh at concentrations of 50 µM slightly reduced UN-SH-SY5Y cell viability (Figure 3a), and this effect was less pronounced in RA-SH-SY5Y cells (Figure 3b).

### 2.3. The Impact of the Vehicle on the Cell Damage Induced by Various Factors in UN- and RA-SH-SY5Y Cells

**Figure 4:**
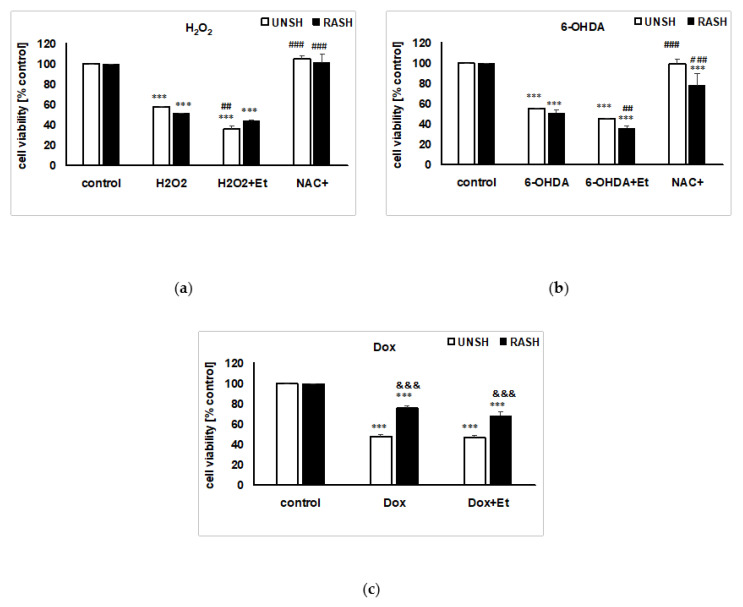
The impact of vehicle (0.7% ethanol) and antioxidant N-acetylcysteine (NAC, 1 mM) on cell damage induced by: (**a**) hydrogen peroxide (H_2_O_2_); (**b**) 6-hydroxydopamine (6-OHDA) and (**c**) doxorubicin (Dox) in UN- (UNSH) and RA-SH-SY5Y (RASH) cells. Statistical analysis was done with two-way ANOVA. Cell viability was measured with WST-1 assay and the data were normalized to the vehicle-treated cells and presented as the mean ± SEM from 4–5 independent experiments with three repetitions. *** *p* < 0.001 vs. vehicle-treated cells; ^##^ *p* < 0.01 and ^###^ *p* < 0.001 vs. toxin-treated cells; ^&&&^ *p* < 0.001 UN- vs. RA-SH-SY5Y cells.

The solvent used to solubilize the flavonols (70% ethanol) when given alone to cell cultures (final concentration 0.7%) for 24 h did not affect the cell viability of UN- or RA-SH-SY5Y cells (data not shown). However, the presence of ethanol exaggerated the cell damage induced by H_2_O_2_ and 6-OHDA in UN- and RA-SH-SY5Y cells (Figure 4a,b). Interestingly, 0.7% ethanol did not change the extent of cell damage induced by the pro-apoptotic factor doxorubicin (Dox) (Figure 4c). Moreover, we confirmed the previous findings that the cell damage induced by H_2_O_2_ or 6-OHDA in SH-SY5Y cells is oxidative stress-dependent [24,25] since the co-treatment with antioxidant (NAC) completely prevented the cell death evoked by these factors (Figure 4a,b).

### 2.4. The Effects of Flavonols on H_2_O_2_-induced Cell Damage in UN- and RA-SH-SY5Y Cells

**Figure 5:**
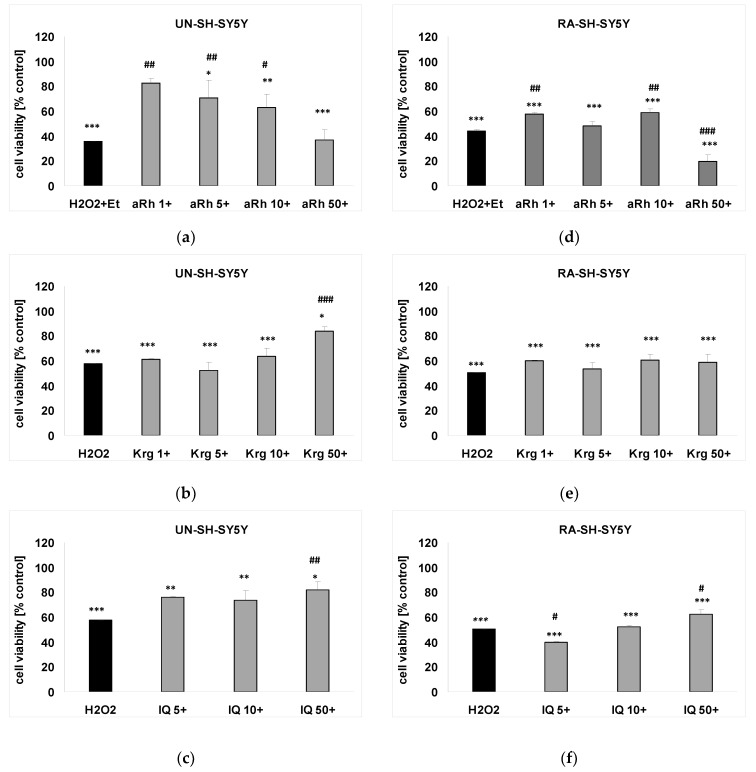
The protective effects of flavonols against hydrogen peroxide (H_2_O_2_)-evoked cell damage in UN- (**a**–**c**) and RA-SH-SY5Y (**d**–**f**) cells. The cells were pre-treated for 30 min with α-rhamnoisorobin (aRh, 1–50 µM; **a**,**d**), kaempferitrin (Krg, 1–50 µM; **b**,**e**) and isoquercitrin (IQ, 5–50 µM; **c**,**f**) followed by 24 h exposure to H_2_O_2_ (0.375 and 0.5 mM for UN- and RA-SH-SY5Y, respectively). Cell viability was measured by WST-1 assay and the data were normalized to the vehicle-treated cells and presented as the mean ± SEM. The data were analyzed by one-way ANOVA. * *p* < 0.05, ** *p* < 0.01, *** *p* < 0.001 vs. the vehicle-treated cells; ^#^ *p* < 0.05, ^##^ *p* < 0.01, ^###^ *p* < 0.001 vs. the H_2_O_2_-treated cells.

aRh almost completely prevented the H_2_O_2_-induced cell damage at concentrations of 1, 5 and 10 µM but not at 50 µM in UN-SH-SY5Y cells (Figure 5a). This protective effect was maintained, although at the lower range (by about 15%), for the concentrations of 1 and 10 µM in RA-SH-SY5Y (Figure 5d). The highest of the tested aRh concentrations (50 µM) slightly potentiated the H_2_O_2_-evoked cell damage (Figure 5d). Krg and IQ at the highest concertation tested demonstrated less pronounced neuroprotection when compared to the effects of aRh in UN-SH-SY5Y cells (Figure 5b,c). In RA-SH-SY5Y cells, except for aRh only, IQ showed beneficial effects (Figure 5f). Krg did not demonstrate neuroprotective activity in this experiment (Figure 5e).

### 2.5. Mechanisms of aRh-, Krg- and IQ-mediated Protection against H_2_O_2_-induced Cell Damage in SH-SY5Y Cells

**Figure 6:**
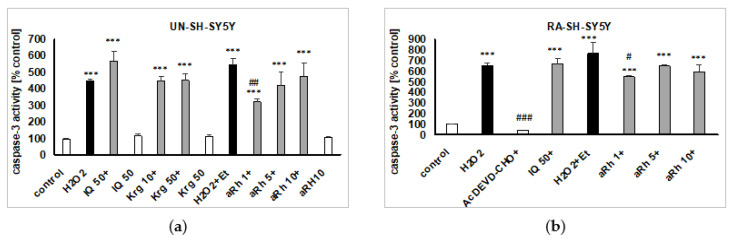
The effects of the tested flavonols against hydrogen peroxide (H_2_O_2_)-induced caspase-3 activity in (**a**) UN- and (**b**) RA-SH-SY5Y cells. The cells were pre-treated for 30 min with isoquercitrin (IQ, 50 µM), kaempferitrin (Krg, 10 and 50 µM) and α-rhamnoisorobin (aRh, 1–10 µM) followed by 9 (UN-) or 18 h (RA-) exposure to H_2_O_2_ (0.375 and 0.5 mM for UN- and RA-SH-SY5Y, respectively). Caspase-3 inhibitor Ac-DEVD-CHO (10 µM) was used as a positive control for the assay. Data were normalized to the vehicle-treated cells and are presented as the mean ± SEM. The data were analyzed by one-way ANOVA. *** *p* < 0.001 vs. the vehicle-treated cells; ^#^ *p* < 0.05, ^##^ *p* < 0.01 and ^###^ *p* < 0.001 vs. the H_2_O_2_ + Et-treated cells.

**Figure 7:**
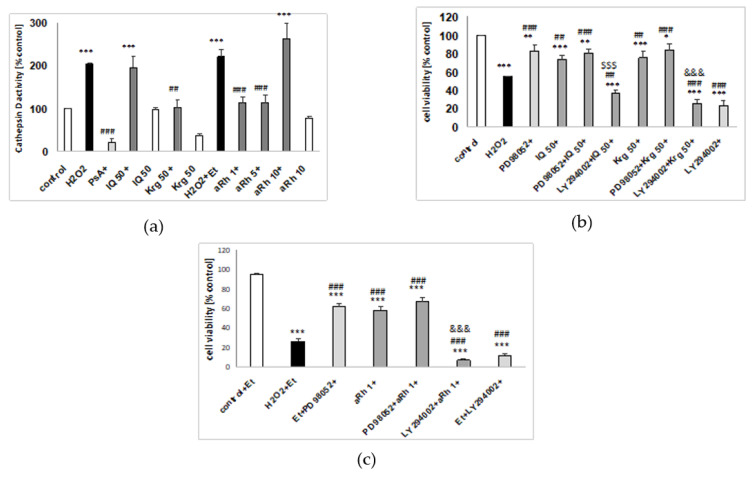
Involvement of cathepsin D, MAPK/ERK1/2 and PI3-K/Akt pathways in neuroprotective action of flavonols: (**a**) The effects of the tested flavonols against hydrogen peroxide (H_2_O_2_)-induced cathepsin D activity in UN-SH-SY5Y cells. The cells were pre-treated for 30 min with isoquercitrin (IQ, 50 µM), kaempferitrin (Krg, 50 µM) and α-rhamnoisorobin (aRh, 1–10 µM) followed by 18 h exposure to H_2_O_2_ (0.375 mM). Cathepsin D inhibitor pepstatin A (PsA, 0.3 µM) was used as a positive control for the assay. (**b**,**c**) The effects of MAPK/ERK1/2 and PI3-K/Akt inhibitors on the flavonol-mediated protection against H_2_O_2_-evoked cell damage. The UN-SH-SY5Y cells were pre-treated for 30 min with the MAPK/ERK1/2 inhibitor PD98052 (10 µM) or the PI3-K/Akt inhibitor LY294002 (10 µM) followed by the next 30 min treatment with IQ (50 µM), Krg (50 µM) or aRh (1 µM) and subsequent exposure to H_2_O_2_ (0.375 mM) for the next 24 h. The data were normalized to the vehicle-treated cells and are presented as the mean ± SEM. The data were analyzed by one-way ANOVA. * *p* < 0.05, ** *p* < 0.01, *** *p* < 0.001 vs. the vehicle-treated cells; ^##^ *p* < 0.01 and ^###^ *p* < 0.001 vs. the H_2_O_2_-treated cells; ^&&&^ *p* < 0.001 vs. H_2_O_2_ + Krg/aRh; ^$$$^ *p* < 0.001 vs. H_2_O_2_ + IQ.

Since our previous studies proved an involvement of caspase-3 and cathepsin D activation in the model of H_2_O_2_-evoked cell damage in SH-SY5Y cells [24,25,26], we examined whether or not the studied flavonols could affect these enzyme activities. IQ (50 µM), Krg (50 µM) and aRh (10 µM), when given alone, did not affect caspase-3 activity in UN- and RA-SH-SY5Y cells after 9 and 18 h of treatment, respectively (Figure 6a,b). From among the tested flavonols, only aRh at the concentration of 1 µM partially attenuated the H_2_O_2_-induced toxic effect in both phenotypes of SH-SY5Y cells (Figure 6a,b). IQ (50 µM), Krg (50 µM) and aRh (10 µM) given alone did not affect cathepsin D activity in UN-SH-SY5Y cells, after 18 h of treatment. Krg (50 µM) and aRh (1 and 5 µM but not 10 µM) in contrast to IQ (50 µM) significantly reduced this enzyme activity (Figure 7a).

Previous studies showed that H_2_O_2_ can evoke prolonged activation of the MAPK/ERK1/2 pathway, which may be detrimental for cell viability. Thus, inhibition of this pathway can cause a protective effect [27,28]. In this study, we confirmed these findings by showing that the MAPK/ERK1/2 inhibitor—PD98052 (10 µM) reduced cell damage induced by H_2_O_2_ or H_2_O_2_ + Et (Figure 7b,c). Since the inhibitor did not change the extent of protection mediated by IQ (50 µM), Krg (50 µM) (Figure 7b) or aRh (1 µM) (Figure 7c), we hypothesized that the studied flavonoids engage the inhibition of MAPK/ERK1/2 in their neuroprotective action. Finally, we employed PI3-K/Akt inhibitor LY294002 (10 µM) to verify the participation of the pro-survival pathway activation in the flavonol-mediated neuronal protection. We not only confirmed our previous results [29] by demonstrating that LY294002 increase cell damage induced by H_2_O_2_ in SH-SY5Y cells (Figure 7b,c), but we also proved that this inhibitor abolished the neuronal protection mediated by IQ, Krg (Figure 7b) and aRh (Figure 7c).

### 2.6. The Effects of Flavonols on 6-OHDA-induced Cell Damage in UN- and RA-SH-SY5Y Cells

**Figure 8:**
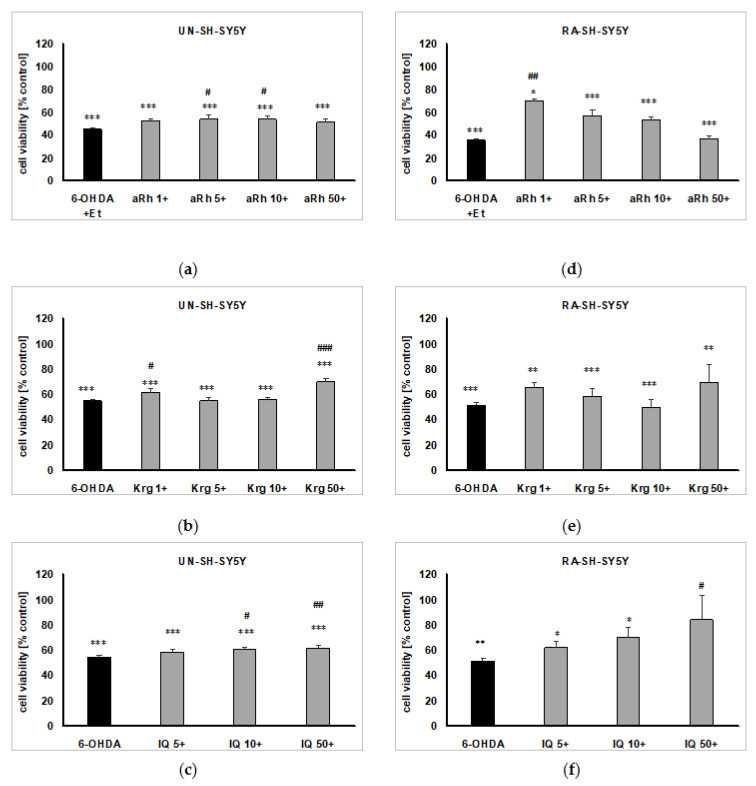
The protective effects of the tested flavonols against 6-hydroxydopamine (6-OHDA)-evoked cell damage in UN- (**a**–**c**) and RA-SH-SY5Y (**d**–**f**) cells. The cells were pre-treated for 30 min with α-rhamnoisorobin (aRh, 1–50 µM; **a**,**d**), kaempferitrin (Krg, 1–50 µM; **b** and **e**) and isoquercitrin (IQ, 5–50 µM; **c**,**f**) followed by 24 h exposure to 6-OHDA (100 and 200 µM for UN- and RA-SH-SY5Y, respectively). The cell viability was measured by WST-1 assay and the data were normalized to the vehicle-treated cells and are presented as the mean ± SEM. The data were analyzed by one-way ANOVA. * *p* < 0.05, ** *p* < 0.01, *** *p* < 0.001 vs. the vehicle-treated cells; ^#^ *p* < 0.05, ^##^ *p* < 0.01 and ^###^ *p* < 0.001 vs. the 6-OHDA-treated cells.

In UN-SH-SY5Y cells, the pretreatment with 5 and 10 µM of aRh (Figure 8a), 1 and 50 µM of Krg (Figure 8b) and 10 and 50 µM of IQ (Figure 8c) exerted a partial but significant protective effect against 6-OHDA-induced toxicity. In RA-SH-SY5Y cells, we found a significant increase in the viability of the 6-OHDA-treated cells pre-treated with 1 µM aRh (Figure 8d) and 50 µM IQ (Figure 8f). We did not notice any protective effect of Krg (1–50 µM) in this model of the cell damage in RA-SH-SY5Y cells (Figure 8e).

### 2.7. The Lack of Protection against Doxorubicin-induced Cell Damage in UN- and RA-SH-SY5Y Cells

Doxorubicin significantly diminished viability of both UN- and RA-SH-SY5Y cells. The cytotoxic effect was less pronounced in the differentiated cells. Pretreatment with the tested flavonols (aRh, Krg and IQ) at a concentration range of 5–50 µM did not counteract Dox-induced cytotoxicity (see Table 1).

## 3. Discussion

Quercetin is the best known and the most frequently investigated plant flavonol [7,30]. Multiple cellular signaling pathways are involved in the neuroprotective effect induced by the compound such as the paraoxonase 2 (PON2) pathway, phosphoinositide 3-kinase (PI3-K) pathway, sirtuin pathway, cyclic AMP response element binding protein (CREB) pathway, mitogen-activated protein kinases (MAPK) pathway, etc. Kaempferol, although more bioavailable than quercetin [31], is less popular as a subject of research. This flavonol and its glycosides are often components of the plant foods, although mostly in minute amounts. According to the “USDA Database for the Flavonoid Content of Selected Foods” [32], capers and saffron are especially rich in kaempferol and its derivatives with the total contents that reach over 200 mg per 100 g of the fresh weight. Vegetables from the Brassicaceae family (arugula, kale, mustard greens) are also a good source of kaempferol (38–59 mg/100 g).

A growing body of research suggests that flavonols are not only active as neuroprotectants in in vitro experimental conditions but are effective in vivo in attenuating deficits in CNS function caused by ischemia, intoxication or age-related dementia and cognitive impairment [33,34,35,36,37]. Isoquercitrin (IQ), used as a reference compound in our study, at the concentration of 100 µM, diminished glutamate-induced toxicity in HT22 hippocampal cells [38,39] and protected rat pheochromocytoma cells (PC12) against 6-OHDA-induced oxidative stress [40], which is consistent with our findings where IQ (50 µM) attenuated cell damage induced by these oxidative stress inducers in human neuroblastoma SH-SY5Y cells. In vivo, the compound demonstrated neuroprotective activity in diabetic neuropathy [41] and in 1-methyl-4-phenyl-1,2,3,6-tetrahydropyridine (MPTP)-induced acute mouse models of Parkinson’s disease [42].

Beneficial effects of kaempferol administration in animal models of Alzheimer’s and Parkinson’s diseases, ischemic stroke, epilepsy, major depressive disorders, and anxiety disorders were recently summarized by Silva dos Santos et al. [6]. Pharmacological research concerning kaempferol glycosides and their action in CNS diseases produced far fewer results. Kaempferol 3-O-rhamnoside (afzelin) and kaempferol 3-O-glucoside (astragalin) prevented neuron damage in a middle cerebral artery occlusion (MCAO)-induced ischemic stroke model in rats [43]. Kaempferitrin (Krg, the compound used in this study), according to González-Trujano et al. [44], may have an anticonvulsant potential. In vitro, afzelin exerted protective effects against Aβ42-induced toxicity in neuroblastoma SH-SY5Y cells.

Krg is the main flavonoid constituent of leaves not only in *M. membranacea* but also in several medicinally used taxa such as *Uncaria guaianensis* (Aubl.) Gmel. [45], *Bryophyllum pinnatum* (Lank.) Oken [46], kenaf [47], *Bauhinia forficata* Link [48], *Cinnamomum osmophloeum* Kaneh. [49], *Justicia spicigera* Schltdl. [44] and others. The kaempferol diglycoside is often accompanied by α-rhamnoisorobin (aRh), afzelin and kaempferol that may be the degradation products, resultant of hydrolysis of the Krg molecule [47,50]. The most known pharmacological activity of Krg is its hypoglycemic effect [48,51,52]. When tested for anti-inflammatory activity, Krg occurred to be less effective than aRh [53,54].

The present study has been aimed at the examination of the neuroprotective effect of Krg and aRh against the oxidative stress-induced cell injury. To the best of our knowledge, the compounds have never been investigated in this respect, until now. Undifferentiated (UN-SH-SY5Y) and retinoic acid-differentiated (RA-SH-SY5Y) neuroblastoma cell cultures were used for the purpose as a reliable experimental model in the initial screening for the potential neuroprotective agents [24,27,29]. Out of the three investigated compounds (IQ, Krg and aRh) only aRh at the concentration of 50 µM, after 48 or 72 h treatment, diminished proliferation of SH-SY5Y cells. After 72 h, decrease in the proliferation of neuroblastoma cells could be additionally observed in the cultures dosed with aRh at 10 µM concentration. The assessment of viability of the UN-SH-SY5Y and RA-SH-SY5Y cells after 24 h treatment with individual flavonols (at 50 µM concentration) revealed that the weak but statistically significant cytotoxic effect of aRh could be observed only in undifferentiated cells. Rho et al. [54] found that aRh is cytotoxic in vitro against B16 melanoma cells (IC_50_—22.9 ± 1.3 µM), as far as we are aware, this is the only study describing cytotoxicity of the compound. Nevertheless, these data point to potential cytostatic and cytotoxic effect of aRh, which may be beneficial in the management of at least certain types of cancers.

All the tested compounds demonstrated cytoprotective activity in the UN-SH-SY5Y cells exposed to H_2_O_2_ (24 h, 0.375 mM). Statistically significant results were observed for IQ and Krg at the highest concentration (50 µM), but aRh was effective at a concentration range of 1–10 µM. At 50 µM, aRh did not show any protection against H_2_O_2_-evoked cell damage, probably due to its own detrimental effect on the viability of the cells. The neuroprotective activity of the investigated flavonols was weaker in the RA-SH-SY5Y cells, which agrees with other cellular neuroprotection studies employing SH-SY5Y cells [55,56]. Only IQ (50 µM) and aRh (1 and 10 µM) demonstrated statistically significant cytoprotective effects against H_2_O_2_-induced oxidative damage in the differentiated neuroblastoma cells, which suggests an involvement of common neuroprotective mechanisms by Krg and RA, and possibly also by aRh and IQ, since the protection in RA-SH-SY5Y was weaker than in UN-SH-SY5Y cells. Among these common mechanisms, activation of pro-survival pathways such as MAPK/ERK1/2 or PI3-K/Akt, decrease in pro-apoptotic (Bax, caspase-3) and increase in anti-apoptotic (Bcl-2) proteins may be mentioned [57,58].

As H_2_O_2_-induced cell damage usually increases caspase-3 and cathepsin D (lysosomal aspartyl protease) activities in SH-SY5Y cells [24,25,26], the next step in our research was an examination on whether the tested compounds affected activities of the enzymes. Only aRh (1 µM) significantly reduced caspase-3 activity in both UN-SH-SY5Y and RA-SH-SY5Y cells. The effect, however, was considerably weaker than that of the caspase-3 inhibitor—Ac-DEVD-CHO (10 µM). The results suggest that the interference with the apoptotic processes in the H_2_O_2_-treated cells may be only partly responsible for the neuroprotective activity of the examined flavonols. IQ (50 µM) did not affect cathepsin D activity, elevated in H_2_O_2_-exposed UN-SH-SY5Y cells. In contrast to IQ, kaempferol glycosides (Krg at 50 µM and aRh at 1–5 µM) reduced the elevated cathepsin D activity back to the control value, although the effect was weaker than that of the pepstatin A (cathepsin D inhibitor).

The involvement of MAPK/ERK1/2 and PI3-K/Akt signaling pathways in the cytoprotective activity of flavonoids is a well-known phenomenon [7,30]. The most selective PI3-K inhibitor, LY294002, was modeled on the structure of quercetin. In our experiments, pretreatment with MAPK/ERK1/2 inhibitor—PD98052 (10 µM) ameliorated toxic effects induced in UN-SH-SY5Y cells by their exposure to H_2_O_2_, which is consistent with the previously published data [27,28]. The effect was comparable to that of IQ (50 µM), Krg (50 µM) and aRh (1 µM). The combined pretreatment with PD98052 and flavonols did not improve viability of the cells when compared to the pretreatment with PD98052 alone, suggesting a common mechanism of action, namely inhibition of MAPK/ERK1/2 pathway. The UN-SH-SY5Y cells pretreated with LY294002 were more susceptible to H_2_O_2_-induced injury, which confirms our previous findings [29]. Combined pretreatment of the cells with LY294002 and flavonoids completely suppressed neuroprotective effect of IQ, Krg and aRh. Taking into consideration that PI3-K/Akt plays a pivotal role in the neuronal survival, the result is not unexpected. In our previous study, however, concomitant use of LY294002 and methyl caffeate did not abolish the cytoprotective effect exerted by the latter compound, pointing to compound-specific response [24].

6-OHDA is a dopaminergic neurotoxin often used to induce animal models of Parkinson’s disease. In this study, it was used to evoke oxidative stress-related cell damage in SH-SY5Y cells. Pretreatment with flavonols caused significant increase of the viability in 6-OHDA-treated cells. UN-SH-SY5Y cells were partially protected against 6-OHDA-evoked damage by aRh (5 and 10 µM), Krg (1 and 50 µM) and IQ (10 and 50 µM). The other plant polyphenol investigated earlier, methyl caffeate, did not protect the undifferentiated neuroblastoma cells against the oxidative damage induced by 6-OHDA. In RA-SH-SY5Y cells, the partial neuroprotective effects toward 6-OHDA-induced injury was achieved with aRh (1 µM) and IQ (50 µM). Krg did not demonstrate significant activity in this experiment. Moreover, the investigated flavonols at a concentration range of 5–50 µM neither protected UN-SH-SY5Y and RA-SH-SY5Y cells against Dox-evoked damage nor potentiated the Dox cytotoxicity. Kaempferol and several of its glycosides, however, can potentiate the cytotoxic action of the antineoplastic agent etoposide as it was shown recently by Kluska and coworkers in HL-60 cells [59].

## 4. Materials and Methods

### 4.1. Chemicals and Solvents

Organic solvents of analytical grade were purchased either from POCh S.A. (Gliwice, Poland) or from Merck (Darmstadt, Germany). Water was purified by a Milli-Q system (Millipore Corp., Bedford, MA, USA). MeOH and MeCN of HPLC grade were purchased from Merck (Darmstadt, Germany). Dulbecco’s Modified Eagle’s Medium (DMEM) and fetal bovine serum (FBS) were from Gibco (Invitrogen, Paisley, UK). WST-1 assay was purchased from Roche Diagnostic (Basel, Switzerland). Caspase-3 (Ac-DEVD-AMC) and cathepsin D (MOCA-Gly-Lys-Pro-Ile-Leu-Phe-Phe-Arg-Leu-Lys(Dnp)-D-Arg-NH2) fluorogenic substrates were obtained from Enzo Life Sciences (New York, NY, USA). All other reagents were from Sigma (Sigma-Aldrich Chemie GmbH, Schnelldorf, Germany). Isoquercitrin (purity > 95% by HPLC) was isolated from flowers of *Xerolekia speciosissima* (L.) Anderb. [60].

### 4.2. General Experimental Procedures

NMR spectra were recorded in CD_3_OD on a Bruker AVANCE III HD 400 (resonance frequency 400.17 MHz) spectrometer (Bruker Corp., Billerica, MA, USA). Optical rotation was determined in MeOH on a PolAAr31 polarimeter (Optical Activity Ltd., Huntingdon, UK). RP-HPLC separations were performed using an Agilent 1200 Series HPLC system (Agilent Technologies Inc., Santa Clara, CA, USA) equipped with a column oven and a diode array detector. Analytical chromatographic separations were conducted at 25 °C, on a Zorbax Eclipse XDB-C18 column 4.6 × 150 mm (Agilent Technologies, Santa Clara, CA, USA). Conventional column chromatography (CC) was conducted using Merck silica gel 60 (0.063–0.2 mm) and Sephadex LH-20 (GE Healthcare, Uppsala, Sweden) Thin layer chromatography (TLC) was performed on Merck silica gel 60 (0.25 mm) precoated plates.

### 4.3. Plant Material

Leaves of *M. membranacea* A. DC. were collected from the Kontum province (Vietnam) and were taxonomically verified by Dr. Nguyen Quoc Binh from the Vietnam Museum of Nature of the Vietnam Academy of Science and Technology (VAST). A voucher specimen (VN-2292) has been deposited in the Institute of Marine Biochemistry VAST in Hanoi.

### 4.4. Isolation and Identification of Phenolic Constituents from Leaves of M. membranacea

Coarsely ground dried leaves of *M. membranacea* (665 g) were extracted with 80% methanol (MeOH, 5 × 4 L) at room temperature. The obtained extracts were concentrated in vacuo to yield 193.5 g of an oily residue. The residue was suspended in water (1 L) and subsequently partitioned with solvents of increasing polarity: n-hexane (5 × 0.4 L), chloroform (CHCl_3_, 6 × 0.4 L), ethyl acetate (EtOAc, 5 × 0.4 L) and n-butanol (BuOH, 5 × 0.4 L). After evaporation of the solvent, the ethyl acetate fraction of the extract (18.22 g) was subjected to CC over silica gel (250 g) using mobile phase gradients of EtOAc in hexane (up to 100% EtOAc) and MeOH in EtOAc (up to 20% MeOH). Fractions (79.6 mg) eluted with hexane-EtOAc (1:1, *v*/*v*) were rich in **1**. The fractions were subjected to ^1^H NMR analysis without further purification. Elution of the column with hexane-EtOAc (1:3, *v*/*v*) gave fractions containing pure **2** and **3** (47.4 and 55.6 mg, respectively). Compound **3** was subsequently recrystallized from MeOH (purity > 95%, by HPLC). Further elution with hexane-EtOAc (1:3, *v*/*v*) yielded fractions containing **4**. The fractions were combined and subjected to further separation by CC on Sephadex LH-20 using initially water (1 L) and subsequently 25% MeOH as an eluent, to give pure **4** (30.1 mg, purity > 95% by HPLC). Fractions eluted from silica gel by 100% EtOAc yielded a mixture of **4** and **5** that was not further separated.

The isolated compounds and components of the mixtures were identified on the basis of their spectral data (UV, ^1^H NMR), their optical activity and their chromatographic behavior.

### 4.5. Assessment of Kaempferol 3,7-di-O-glycosides Contents in Extracts from the Leaves of M. membranacea

The contents of kaempferol 3-*O*-α-arabinopyranoside-7-*O*-α-rhamnopyranoside (**5**) and Krg (**4**) in 70% methanol extracts and in water infusions prepared from the leaves of *M. membranacea* were estimated by the routine HPLC/DAD method described earlier [61], with a reference to a calibration curve, based on UV detection at 340 nm, prepared for the pure Krg (purity > 95%).

### 4.6. SH-SY5Y Cell Culture

The human SH-SY5Y neuroblastoma cells (ATCC, passages 4–19) were cultured as described previously [26]. Cells were maintained in DMEM supplemented with 10% heat-inactivated FBS and 1% penicillin/streptomycin solution, at 37 °C, in an atmosphere containing 95% air and 5% CO_2_ with saturated humidity. After reaching an 80% confluence, cells were counted using Bürker chamber and seeded at a density of 3 × 10^4^ and 1 × 10^6^ cells per well into 96- and 6-well plates, respectively. Cell differentiation to neuronal phenotype was performed with the cells plated at a half of the densities mentioned above and cultured in a medium supplemented with retinoic acid (RA, 10 µM), for 6 days, with medium exchange every two days. One day prior experiments the culture medium for both cell phenotypes (UN-SH-SY5Y and RA-SH-SY5Y) was replaced with DMEM containing 1% FBS and 1% penicillin/streptomycin solution in order to limit cell proliferation.

### 4.7. Cell Treatment

First, a putative impact of aRh, Krg and IQ on cell proliferation was tested in SH-SY5Y cells seeded at density 1 × 10^4^ cells/well in a 96-well plate format in a cell growing medium containing 10% FBS. Twenty four hours after plating, cells were treated with aRh (1, 5, 10 and 50 μM), Krg (5, 10, 50 μM) and IQ (5, 10, 50 μM) for 48 and 72 h. To assess biosafety of the compounds, UN-SH-SY5Y and RA-SH-SY5Y cells were treated for 24 h with aRh (50 μM), Krg (50 μM) and IQ (50 μM) in experimental cell culture medium (with 1% FBS). For testing the neuroprotective potency, the UN- and RA-SH-SY5Y cells were pre-treated for 30 min with aRh (0.5–50 μM), Krg (5–50 μM) or IQ (5–50 μM), followed by 24 h exposure to H_2_O_2_ (0.375 mM and 0.5 mM for UN- and RA-SH-SY5Y cells, respectively). Moreover, we tested the effect of these flavonols in the model of cell damage induced by 6-hydroxydopamine (6-OHDA; 100 and 200 μM for UN- and RA-SH-SY5Y cells, respectively) or doxorubicin (Dox; 0.375 and 0.5 μM for UN- and RA-SH-SY5Y cells, respectively). The antioxidant N-acetylcysteine (NAC, 1 mM) was used as a positive control for oxidative stress-induced cell damage models (H_2_O_2_ or 6-OHDA). The effective concentrations of particular cell damaging factors (H_2_O_2_, 6-OHDA or Dox,) were established in our previous studies, where these factors reduced cell viability by about 50% [24]. For mechanistic studies, inhibitors of MAPK/ERK1/2, PD98052 (10 μM) and PI3-K, LY294002 (10 μM) were given 30 min before the particular flavonol exposure.

IQ (10 mM), aRh (5 mM) and Krg (5 mM) stock solutions were prepared in 70% ethanol, aliquoted and stored at –20 °C. The final solutions of IQ, Krg and aRh were prepared in distilled water, 35% ethanol and 70% ethanol, respectively. Ac-DEVD-CHO (10 mM), pepstatin A (PsA, 10 mM), PD98052 (10 mM), and LY294002 (10 mM) stock solutions were prepared in DMSO and Dox (5 mM) in distilled water. The H_2_O_2_ (25 and 50 mM) stock solutions were prepared from stabilized 30% hydrogen peroxide diluted in distilled water. The 6-OHDA (10 mM) stock solution was prepared immediately before use in distilled water. All agents were added to the culture medium at the indicated concentrations under light limited conditions. Each experimental set of the control cultures was supplemented with the appropriate vehicles, and the solvent was present in cultures at a final concentration of 0.1%.

### 4.8. Cell Proliferation Assay

The effect of studied phytochemicals on cell proliferation in UN-SH-SY5Y cells was measured by 3-[4,5-dimethylthiazol-2-yl]-2,5-diphenyltetrazolium bromide (MTT) assay as described previously [24]. The absorbance of the probes was measured after 30 min from substrate addition at 570 nm with a microplate reader Infinite M200 PRO (Tecan, Männedorf, Switzerland). Data were normalized to the vehicle-treated cells and are expressed as a percentage of the control ± SEM established from 2 independent experiments with 3 replicates.

### 4.9. Cell Viability Assay

For the biosafety and neuroprotection assessments of the tested compounds, the cell viability of UN- and RA-SH-SY5Y cells, after particular treatments, was measured by the WST-1 assay according to supplier’s instruction (Roche Diagnostic, Basel, Switzerland). The absorbance of the probes was measured 30 and 60 min after substrate addition at 440 nM (WST-1 assay) with a microplate reader Infinite M200 PRO (Tecan). Data were normalized to the vehicle-treated cells and are expressed as a percentage of the control ± SEM established from 3–5 independent experiments with 3–5 replicates.

### 4.10. Caspase-3 Activity Assay

The cells were grown in the 6-well format and were pretreated for 30 min with flavonols followed by 9 h or 18 h exposure to H_2_O_2_ (UN- and RA-SH-SY5Y cells, respectively). The caspase-3 activity was measured in cell lysates using fluorogenic substrate Ac-DEVD-AMC (50 μM) as described previously [24]. Caspase-3 inhibitor, Ac-DEVD-CHO (10 μM) was used to verify the assay specificity. The data (expressed as the mean relative fluorescence units, RFU) were normalized to the protein level (measured by BCA method), calculated as a percent of vehicle-treated cells and presented as the mean ± SEM from 3–5 separate experiments with 2 repetitions each.

### 4.11. Cathepsin D Activity Assay

The cells were grown in the 6-well format and were pretreated for 30 min with flavonols followed by 18 h exposure to H_2_O_2_ in UN-SH-SY5Y cells. Cathepsin D activity in cell lysates was measured using a fluorogenic substrate AMC-Gly-Lys-Pro-Ile-Leu- Phe-Phe-Arg-Leu-Lys(Dnp)-D-Arg-NH_2_ as described previously [24]. PsA (0.3 μM) was used as a positive control for the assay. Cathepsin D activity was normalized to the protein level (measured by BCA method), calculated as a percent of vehicle-treated cells and presented as the mean ± SEM from 4 separate experiments with 2 repetitions each.

### 4.12. Statistical Analysis

Data were analyzed using the Statistica software [62]. The analysis of variance (one- or two-way ANOVA) and post hoc Duncan test for multiple comparisons were used to show statistical significance with assumed *p* < 0.05.

## 5. Conclusions

Kaempferitrin and α-rhamnoisorobin showed protective effect in certain in vitro models of oxidative stress-induced neurotoxicity (H_2_O_2_, 6-OHDA) in both undifferentiated and differentiated SH-SY5Y cells. The activity seems to be connected with the activation of the prosurvival PI3-K/Akt pathway and inhibition of the MAPK/ERK1/2 pathway. The distinctive feature of the examined kaempferol derivatives in the H_2_O_2_-evoked model of neurotoxicity seem to be cathepsin D inhibition that was not observed for isoquercitrin. The only compound that induced statistically significant inhibition of caspase-3 activity in the experimental model applied in the study was α-rhamnoisorobin. The kaempferol monorhamnoside demonstrated neuroprotective activity in considerably lower concentrations than isoquercitrin and kaempferitrin. The flavonols were ineffective in the preventing of doxorubicin-induced damage in neuroblastoma cells. Altogether, our findings from different cell damage models of neuronal-like cells clearly show the flavonoid compound-dependent response with the engagement of common (MAPK/ERK1/2, PI3-K/Akt) and different (caspase-3 and cathepsin D) mechanisms in their neuroprotective action.

## Figures and Tables

**Table 1:** The effects of the investigated flavonols against doxorubicin (Dox)-evoked cell damage in neuroblastoma (UN- and RA-SH-SY5Y) cells.

	UN-SH-SY5Y	RA-SH-SY5Y
Control	100.00 ± 0.00	100.00 ± 0.00
Dox	48.15 ± 1.15 ***	76.62 ± 2.44 ***
IQ 5+	43.40 ± 3.73 ***	72.24 ± 1.64 ***
IQ 10+	51.01 ± 4.86 ***	74.20 ± 4.23 ***
IQ 50+	53.60 ± 6.32 ***	72.63 ± 4.26 ***
Krg 5+	49.97 ± 4.49 ***	76.99 ± 1.35 ***
Krg 10+	49.96 ± 2.80 ***	78.96 ± 1.69 ***
Krg 50+	51.13 ± 4.32 ***	73.94 ± 1.85 ***
Dox + Et	46.81 ± 2.28 ***	68.38 ± 3.34 ***
aRh 5+	51.25 ± 4.32 ***	73.38 ± 0.80 ***
aRh 10+	40.32 ± 2.63 ***	70.47 ± 0.61 ***
aRh 50+	41.64 ± 4.10 ***	66.77 ± 2.79 ***

The cells were pre-treated for 30 min with vehicle (Et, 0.7% ethanol), isoquercitin (IQ, 5–50 µM), kaempferitin (Krg, 5–50 µM) or α-rhamnoisorobin (aRh, 5–50 µM) followed by 24 h exposure to doxorubicin (Dox, 0.375 and 0.5 µM for UN- and RA-SH-SY5Y, respectively). Cell viability was measured by WST-1 assay. The data were normalized to the vehicle-treated cells and are presented as the mean ± SEM. The data were analyzed by one-way ANOVA. *** *p*<0.001 vs. vehicle-treated cells.

## Data Availability

The raw data that support the findings of this study are available from the authors, [D.J., J.M., A.S.], upon reasonable request.

## Abbreviations

Akt	Protein kinase B
aRh	α-Rhamnoisorobin (kaempferol 7-*O*-α-rhamnoside, PubChem CID 25079965)
Dox	Doxorubicin
ERK1/2	Extracellular signal-regulated kinase 1/2
IQ	Isoquercitrin (quercetin 3-*O*-*β*-glucoside, PubChem CID 5280804)
Krg	Kaempferitrin (kaempferol 3,7-di-*O*-α-rhamnoside, PubChem CID 5486199)
MAPK	Mitogen-activated protein kinase
6-OHDA	6-Hydroxydopamine
PI3-K	Phosphoinositide 3-kinase
RA	Retinoic acid
ROS	Reactive oxygen species
